# Demonstration of extrinsic chirality of photoluminescence with semiconductor-metal hybrid nanowires

**DOI:** 10.1038/s41598-019-41615-1

**Published:** 2019-03-25

**Authors:** Teemu Hakkarainen, Emilija Petronijevic, Marcelo Rizzo Piton, Concita Sibilia

**Affiliations:** 1 Optoelecronics Research Centre, Physics Unit, Tampere University, Korkeakoulunkatu 3, FI-33720 Tampere, Finland; 2 Department S.B.A.I., Sapienza Università di Roma, Via A. Scarpa 14, I-00161 Rome, Italy; 3 Departamento de Física, Universidade Federal de São Carlos, CP 676 São Carlos, São Paulo, Brazil

## Abstract

Chiral optical response is an inherent property of molecules and nanostructures, which cannot be superimposed on their mirror images. In specific cases, optical chirality can be observed also for symmetric structures. This so-called extrinsic chirality requires that the mirror symmetry is broken by the geometry of the structure together with the incident or emission angle of light. From the fabrication point of view, the benefit of extrinsic chirality is that there is no need to induce structural chirality at nanoscale. This paper reports demonstration extrinsic chirality of photoluminescence emission from asymmetrically Au-coated GaAs-AlGaAs-GaAs core-shell nanowires fabricated on silicon by a completely lithography-free self-assembled method. In particular, the extrinsic chirality of PL emission is shown to originate from a strong symmetry breaking of fundamental HE_11_ waveguide modes due to the presence of the asymmetric Au coating, causing preferential emission of left and right-handed emissions in different directions in the far field.

## Introduction

Chirality is an intrinsic property of structure that cannot be superimposed on its mirror image^1^. Such lack of mirror symmetry is found in DNA, proteins, sugars, viruses, and amino acids among other important molecules and building blocks of life. The interaction of chiral molecules with light is different for left and right-handed circular polarizations^2^. This so-called circular dichroism (CD) of chiral molecules is typically observed in light absorption^3^. The optical response of chiral molecules can be mimicked with artificial nanostructures with chiral shape^4^ fabricated using nanolithography^5^, focused ion beam-induced deposition^6^ and other nanofabrication methods. On the other hand, chiral optical response can be accessed also with structures that themselves are not chiral, given that specific conditions are fulfilled. This extrinsic optical chirality requires that the geometry of the structure and, for example, the incidence angle of light together break the mirror symmetry, as it has been demonstrated for absorption^7^, transmission^8^, reflection^9^, optical activity^10,11^ and non-linear response^12^. The benefits of extrinsic chirality include easier fabrication and freedom of design, as it is not required to induce structural chirality at nanoscale. Furthermore, same structure can be used to interact with both circular polarizations by changing incidence angle or orientation of the structure. The chiral optical response can be extended also to light emission. Luminescent chiral molecules can emit preferentially either left-handed or right-handed circular polarization depending on their handedness^13,14^, while in semiconductors circular polarized photoluminescence (PL) is associated with different spin states of charge carriers^15^. There are only few reports of extrinsic chiral effects in light emission. Yokoyama *et al*. reported circular polarization dependence of excitation in photoluminescence from carbon nanotubes placed on nanostructured silicon surface^16^, while Yan *et al*. used extrinsic chirality provided by an array of metal nanoantennas for splitting left and right-handed circular polarizations of fluorescence from achiral fluorophores in different direction in the far field^17^.

In the application point of view, the semiconductors have significant benefits in the integration with the existing optoelectonic and microelectronic technologies. In particular, direct bandgap III-V semiconductor nanowires (NW) of high optical and electrical quality can be grown directly on silicon substrates by Au-catalyzed^18,19^ and self-catalyzed^20^ vapor-liquid-solid method^21^. Owing the their geometry and high refractive index, semiconductor NWs can effectively confine and manipulate electromagnetic fields in the visible and near infrared wavelengths. These properties are essential for several NW device concepts^22–26^, where coupling of the light to the resonant modes of the nanostructure^27,28^ can tailor the light absorption^29,30^, extraction and directionality of emission^31–35^, as well as stimulated emission^36,37^. More recently, we have shown that asymmetrically Au-coated NWs exhibit extrinsic chirality and CD in absorption at the wavelengths where the incident light is coupled to the waveguide modes^38^.

Here, we demonstrate extrinsic chirality of PL emission from hybrid semiconductor-metal structures consisting of asymmetrically Au-coated GaAs-AlGaAs core-shell NWs grown on silicon substrates. The chiral PL emission from the GaAs core with linear polarized excitation originates from a strong symmetry breaking of HE_11_ modes due to the presence of Au on three of the six (111) sidewalls, leading to a preferential directionality of left and right-handed circular polarizations in different angles in the far field. A unique property of this concept is that the intrinsic waveguiding properties of the NWs are modified with the external Au layer to provide extrinsic chirality of light emission. Furthermore, they are fabricated with a completely lithography-free, self-assembled technique and potentially also allow electrical injection for LED operation by incorporating a PN-junction in the NW, thus providing a chiral light manipulation platform for applications spanning from quantum information technology to biology and chemistry.

## Experimental Methods

**Figure 1:**
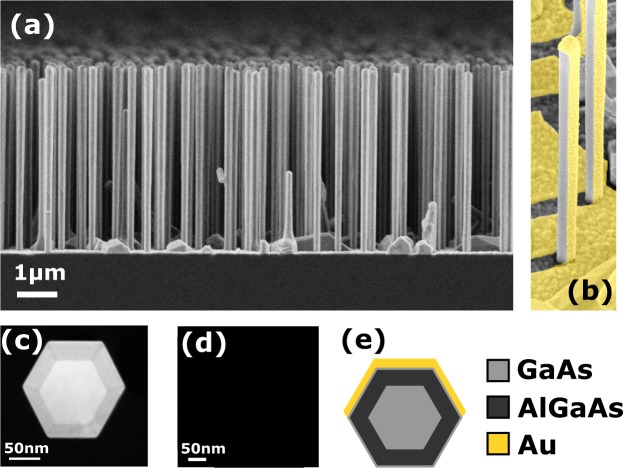
Structural details of the investigated NWs. (**a**) SEM edge view the sample with NWs 4690 ± 80 nm long, of the overall diameter 197 ± 9 nm. (**b**) False color SEM image of the NWs after Au deposition. (**c**) Cross-sectional dark-field TEM micrograph showing GaAs core, AlGaAs shell and GaAs supershell. (**d**) Cross-sectional TEM image of the NW asymmetrically covered by Au. (**e**) Cross-sectional sketch of the NW materials.

The investigated sample consists of vertically standing NWs grown by molecular beam epitaxy on Si(111) wafers using lithography-free Si/SiOx patterning technique for defining the nucleation sites^39^. This technique has been recently proven to provide vertical NW forests with highly uniform dimensions^40,41^. Consequently, the optical response of the ensemble is strongly governed by the single NW response simulated assuming the mean values of the NW dimensions^30,38^. The NW structure includes a GaAs core, an AlGaAs shell and an GaAs supershell. The overall diameter of the NW is *D* = 197 ± 9 nm, which includes a 27.7 nm thick AlGaAs passivation shell, and a 5.5 nm GaAs supershell. The length is 4690 ± 80 nm and density around 1 × 10^8^ cm^−2^. These structural parameters are mean values from statistical analysis of a large number of NWs^30^. Figure 1a shows an edge-view scanning electron microscope (SEM) image of the NWs investigated in this work. The growth of the GaAs-AlGaAs-GaAs core-shell heterostructure NWs is reported in ref.^39^. The semiconductor-metal hybrid structures were obtained by growing a thin Au layer on the NWs using electron beam evaporation. The NW sample was tilted in a 14° angle in order to obtain the asymmetric structure presented in Fig. 1b. The Au flux angle was chosen based on the average length and nearest neighbor statistics of the NWs, thus minimizing the shadowing effects as shown in the Supplementary Fig. S1. The sample orientation with respect to the Au flux and the deposition time were chosen in such way that one of the NW side facets would get nominally a 20 nm thick and the two side adjacent facets a 10 nm thick Au layer. Figure 1d shows a cross-sectional transmission electron microscopy (TEM) micrograph of the resulting metal-semiconductor structure while the complete layer structure is schematically presented in Fig. 1e.

**Figure 2:**
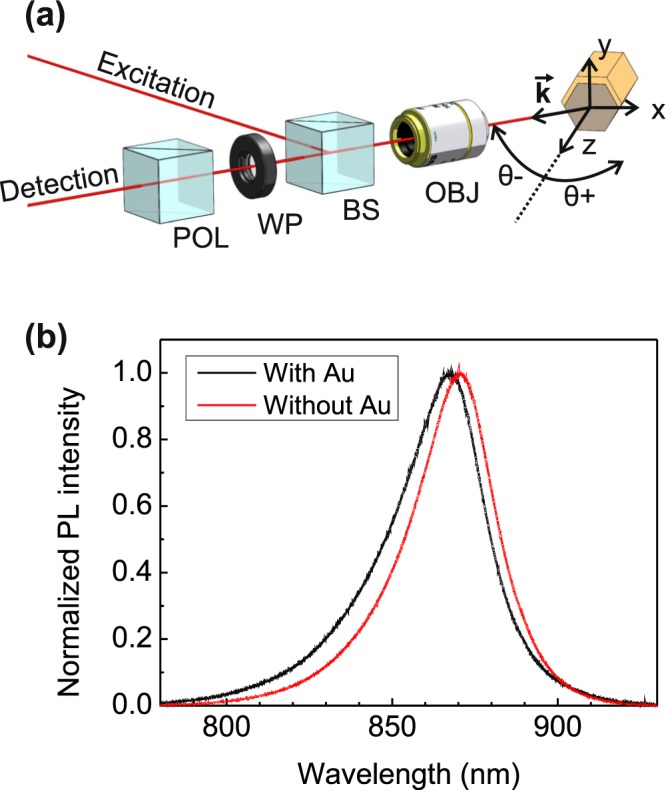
(**a**) Polarized detection set-up: NWs are tilted in *xz*-plane by angle *θ* with respect to the detection line consisting of a microscope objective (OBJ), a rotating quarter-wave plate (WP), a fixed linear polarizer (POL), and a detection part which includes a spectrometer and a CCD camera. (**b**) Unpolarized photoluminescence spectra for NWs with and without Au at *θ* = 0°.

In the optical experiments, the Au-coated NWs are excited by a 640 nm diode laser operating in continuous wave. The NWs were tilted by an angle *θ* in the *xz*-plane with respect to the optical axis represented by the wave vector **k**, as shown in Fig. 2a. The emitted light was collected from the far field with a 10X magnifying objective (*NA* = 0.22). The circular polarization of the photoluminescence (PL) emission was resolved using a rotating broadband quarter-wave plate and a fixed linear polarizer. The PL signal was dispersed with a 750 mm spectrograph equipped with a 1200 l/mm grating and detected with a TE-cooled CCD array. A detailed description of the experimental setup is presented in Supplementary Fig. S2. All measurements were carried out at room temperature. Both the as-grown and Au-coated NWs exhibit a typical GaAs band edge PL emission around with a peak at around 870 nm, as shown in Fig. 2b. A slight blueshift is observed in the presence of Au, most likely due to effect of Au on band bending^42^ and consequent change of PL transition energy. In the following we will focus on the polarization properties of the light emitted at 868 nm. It should be noted that, while use of circular polarized excitation affects the absorption efficiency of the asymmetrically Au-coated NWs^38^ and thus influence the generation rate of the photoexcited electron-hole pairs, it does not affect the extrinsic chirality of the PL emission, as shown in the Supplementary Fig. S3. Therefore, the optical experiments discussed in this work were carried out using linear polarized excitation.

## Experimental Results

An achiral nanostructure can exhibit chiral optical response if the light wave vector **k**, the vector pointing in the direction of anisotropy of the structure, and the surface normal of the sample do not lie in the same plane^7,12^. This phenomenon is called extrinsic chirality, because the breaking of mirror symmetry is not an intrinsic property of the material, but a property of the experiment and the sample together. In the Au-coated NW samples, the extrinsic chiral behavior can observed given a proper orientation of the NW and its Au-coated sides with respect to the light to be absorbed or emitted: the average anisotropy of the structure is in the *y*-direction, the NW axis (the surface normal) is in the *z*-direction, while the wave-vector of the emitted light lies in *xz*-plane in such way that **k** is not parallel with the *z*-axis. In the actual experiment we tilt the sample stage in such way that it equals to rotation of nanowire axis around the *y*-axis in the geometry presented in Fig. 2a. The amount rotation is measured by the angle *θ* with respect to the k direction. The quarter-wave plate then scans the polarization, with 45° representing right-handed and 135° left-handed circular polarization.

**Figure 3:**
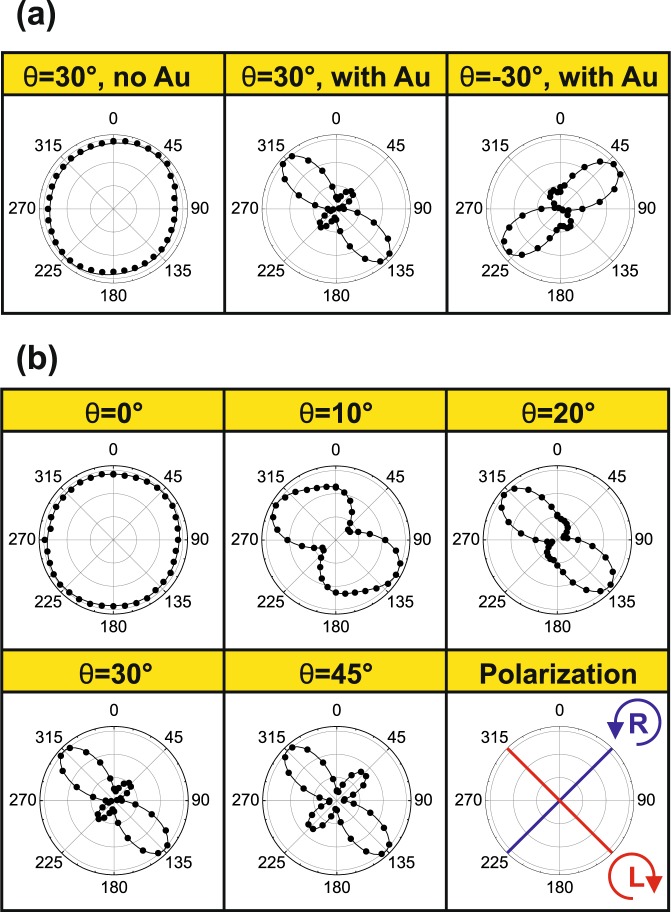
Chirality of photoluminescence emission with polarized detection presented as polar plots of normalized intensity versus *λ*/4 waveplate angle: (**a**) for NWs without Au and for NWs with Au for positive and negative tilt angles (*θ*). (**b**) Different tilt angles for NWs with Au. The dots represent experimental data and the continuous lines are double sinusoidal fits. The radial scale in each plot ranges from 0.7 to 1.02. The last panel in (**b**) presents the WP angles corresponding the right-handed (R) and left-handed (L) polarizations.

In Fig. 3 we present polar plots of the detected PL intensity as a function of the wave plate angle. As shown in Fig. 3a, the NWs without Au do not show any difference between the left and right-handed polarizations, as expected from the lack of anisotropy between the *x* and *y*-directions. The sample with Au shows a clear left-handed polarization with an intense lobe at 135° and significantly smaller intensity at 45° for *θ* = 30°, and the polarization changes to right-handed when the sample is rotated to *θ* = −30°. It should be noted that PL intensity includes a linear polarized contribution that is summed in the circular polarized signal, and therefore polar plots in Fig. 3a exhibit some degree of ellipticity. Nevertheless, these observations clearly manifest extrinsic chiral behavior^10^. Next, we investigate the influence of the tilt angle *θ* on the polarization of the Au-coated NWs (Fig. 3b). In case of *θ* = 0°, all three vectors discussed before lie in the same plane, and therefore we do not observe the extrinsic chirality. For tilt angles *θ* > 0 we observe left-handed polarization which increases as a function of *θ*, reaches maximum at 20°, and then decreases again at larger tilt angles. The extrinsic chirality in this system is therefore detected as a difference in circular polarization of the PL emission for different emission angles in the far field hemisphere, meaning that the Au layer can control the direction and the handedness of the emission from GaAs core. The degree of polarization and its dependence on the tilt angle *θ* will be discussed on a more quantitative level in the following as we introduce a theoretical model for PL emission from the Au-coated NWs.

## Model

**Figure 4:**
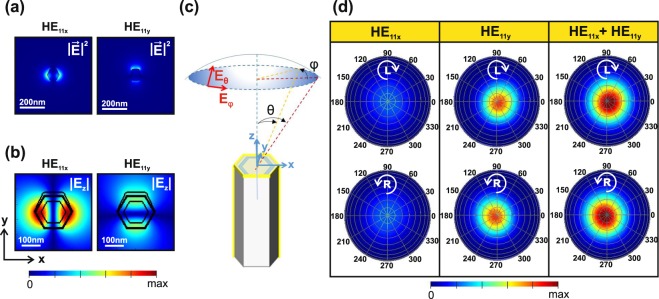
Electric field intensities (**a**) |**E**|^2^ and (**b**) |**E**_**z**_| for HE_11x_ and HE_11y_ modes supported at 868 nm. (**c**) Illustration of the far field simulation geometry. (**d**) Simulated left-handed (L) and right-handed (R) polarizations of the far field intensities emitted from HE_11x_ and HE_11y_ modes. The tilt axis *θ* in (**d**) ranges from 0° to 90° with 10° steps while the azimuthal axis *φ* presents a full rotation around the NW axis.

The coupling of emitted light to the NW modes and its influence on CD was investigated using two numerical packages from Lumerical^43^. First, we use MODE solver to calculate complex refractive indices and field profiles of the guided modes supported in the PL peak wavelength (see Supplementary Info for the details). The NWs investigated in this work have an average diameter of 197 nm and they support the fundamental HE_11_-like mode at emission wavelength of 868 nm. Au strongly breaks the degeneracy of this mode between the *x* and *y*-directions, leading to a significant difference between properties of the HE_11x_ and HE_11y_ modes, as show in Fig. S7 and in Supplementary Information. The density of the investigated NWs is 1 × 10^8^ cm^−2^ and they have average nearest neighbor distance of 500 nm (Supplementary Fig. S6). In ref.^30^ we investigated the resonant absorption properties of similar NWs without Au and showed that the NW ensemble exhibits modal properties governed by single NW dimensions, which can be accurately described by the statistical average values due to the NW size uniformity. It should also be noted that mode spreading is less pronounced in Au-coated than in bare NWs (Supplementary Fig. S7). Consequently, as shown in Fig. 4a, the HE modes that are weakly guided on the NW borders have spatial spreading to at most 180 nm from the center of the NW, which is significantly less than the nearest neighbor distance. Therefore, we can exclude the interaction between the NW neighbors in the following analysis. In Fig. 4b the *E*_*z*_ component along the polarization direction for HE_11y_ mode is higher in the vicinity of Au and asymmetric in the core, which provides the asymmetry required for extrinsic chirality.

Next, we use the finite 3D Finite Difference Time Domain (FDTD) solver to monitor the far field from the NW excited by the HE_11x_ and HE_11y_ modes at 868 nm. The polarization of the emission in a specific angle in the far field can be resolved by taking complex tangential components *E*_*φ*_ and *E*_*θ*_, and multiplying them by Jones transfer matrices of the quarter-wave plate with rotation angles corresponding to the left and right-handed polarizations, and the linear polarizer, thus mimicking the experiment. In Fig. 4c the far field angles (*θ*,*φ* = 0°) and (*θ*,*φ* = 180°) correspond to the experimental tilting of *θ* and –*θ*, respectively, as *φ* represents rotation around the NW axis. The emission from finite NWs is governed by the coupling of the emission centers to the modes, and it is usually investigated by placing a single dipole source inside the NW^28,34^ and calculating the far field contribution of that simulation. For intrinsically isotropic systems, such as zincblende (ZB) one needs to superpose results from simulations with three dipole orientations^44^. This would be the case also with the ZB NWs investigated in this work, where the transitions produce both radially and axially polarized intensity, as proved by the linear PL measurements (Supplementary Fig. S5). Moreover, one would need to perform several simulations to average the contributions from a large number dipole positions across the NW volume to the far field polarization. This is required because of the different spacing of the antinodes of the HE_11x_ and HE_11y_ modes along *z*-direction due to their different *λ*_*z*_, and the asymmetry of the field components in *xy*-cross-section. As the Au layer makes such averaging complicated and time-consuming, here we show that the mode solver and FDTD can be used in consecutively to model the extrinsic chirality of PL from the Au-coated NWs by performing only two simulations. Namely, the two modal fields, which contain complete complex electromagnetic field information in the NW *xy*-cross-section, are first calculated with the mode solver, and then imported as sources that excite the NW in FDTD solver. The intensity is then calculated for the specific quarter-wave plate angle at each point in the far field (see more details in Supplementary Information). As show in Fig. 4d, the results from the excitation with HE_11x_ mode show less intensity with respect to HE_11y_, which is in agreement with its higher losses. Moreover, HE_11x_ mode does not contribute to CD (Supplementary Fig. S8). However, HE_11y_ has a significant difference between the left and right-handed intensities at oblique angles. The final result for the left and right-handed intensities is obtained by summing the contribution from both modes. Again, we see a clear difference between the left and right-handed intensities at oblique angles for the sum of the HE_11x_ and HE_11y_ modes, but it should be stressed that it arises solely from the HE_11y_ asymmetry. It should also be noted that these modes almost degenerate in the case of a bare NW, and therefore no circular polarization is observed for the NWs without Au (Supplementary Fig. S9).

**Figure 5:**
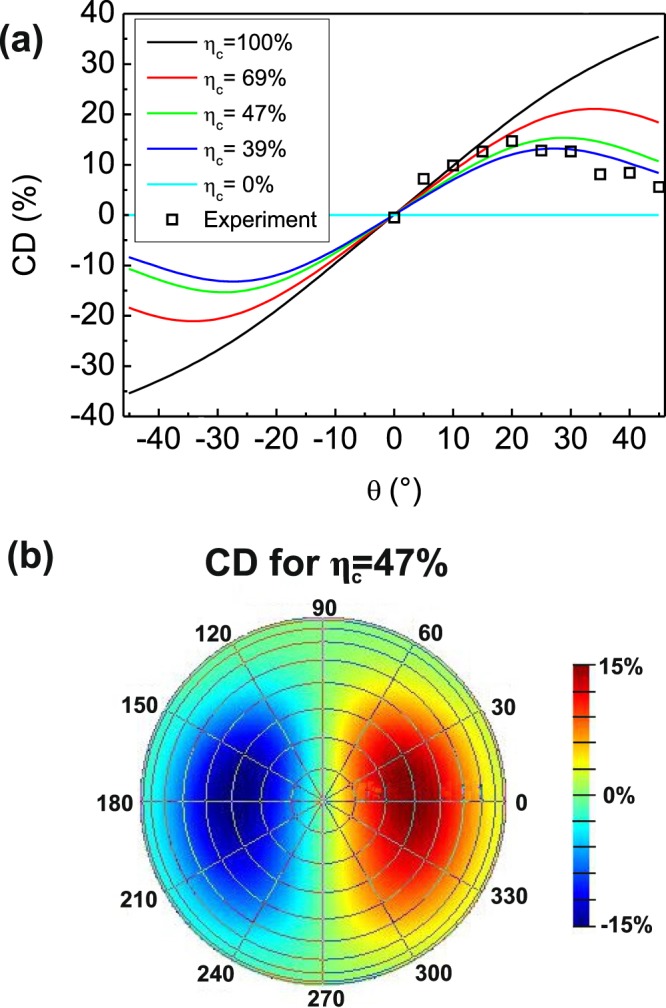
(**a**) The dependence of CD on tilt angle *θ*. The result of the far field model is fitted to the experimental data by introducing a coupling efficiency factor *η*_*c*_ as a fitting parameter. (**b**) Far field CD map *η*_*c*_ = 47%, which produces the best fit. The tilt axis *θ* in (**b**) ranges from 0° to 90° with 10° steps.

We can further introduce a figure of merit for CD in the emission:

1 $$CD[ \% ]=\frac{{I}_{L}-{I}_{R}}{{I}_{L}+{I}_{R}}\cdot 100,$$

where *I*_*R*_ and *I*_*L*_ are the left and right-handed intensities measured at the quarter-wave plate angles of 45° and 135°, respectively. These intensities contain contributions from both modes, and from the unpolarized, isotropic background that can be modelled with a Lambertian source^35^ representing the uncoupled part PL emission, the contribution of which effectively lowers CD (see the complete definition of CD in Supplementary Information). We use the intensity of the Lambertian source as the only fitting parameter and present the results for different values of the coupling efficiency of the NW emission to HE_11_ modes defined as:

2 $${\eta }_{c}=\frac{{I}_{HE11x}+{I}_{HE11y}}{{I}_{HE11x}+{I}_{HE11y}+{I}_{Lambert}},$$

where *I*_*HE11x*_, *I*_*HE11y*_ and *I*_*Lambert*_ represent the total power emitted to the upper half-space by HE_11x_, and HE_11y_ sources exciting the NW, and uncoupled intensity modelled with the Lambertian source, respectively. In Fig. 5a we show CD as a function of tilt angle *θ* for different values of *η*_c_ at *φ* = 0°. As expected, strong coupling leads to the increase of CD due to the high contribution of HE_11y_ to chirality. Lower coupling follows the experimentally measured CD, with *η*_*c*_ = 47% giving the best fit which reproduces the experimentally observed maximum CD of 15%. In Fig. 5b the far field CD for this coupling proves the concept of extrinsic chirality: it is equal to 0 for all *θ* at *φ* = 90° and *φ* = 270°, as no breaking of symmetry takes place in such configuration, and it inverts the sign with the inversion of *θ* where CD exists. The presented model also provides means for further optimization for obtaining stronger polarization. Even larger values of CD could be achieved by (i) increasing the symmetry breaking between the *x* and *y*-directions, (ii), enhancing the coupling of the PL emission to the modes (e.g. by making the NW core thicker to increase the mode overlap with the emitting region), or (iii) confining the emitting material in the form of quantum dots^25^ or superlattices^45^ at the particular positions that lead to stronger CD (e.g. in the antinode of the HE_11y_, as shown in Supplementary Information).

## Conclusions

In summary, we have demonstrated extrinsic chirality of PL emission from asymmetrically Au-coated GaAs-AlGaAs-GaAs core-shell NWs fabricated using a completely lithography-free self-assembled technique. Splitting of left and right-handed polarizations in different directions in the far field was measured by circular polarization dependent detection for different sample tilt angles. The maximum value of CD = 15% was obtained at 20° tilt. The chiral luminescence from the semiconductor-metal hybrid system was modelled using a new approach based on solving the HE_11_ modes supported at the GaAs emission wavelength, and consecutively, using the modes as sources in FDTD simulation of the polarization response in the far field. From this theoretical investigation, we found out that the extrinsic chiral response of the investigated structure arises from the HE_11y_ mode due to the symmetry breaking provided by the metal. Furthermore, it was shown that 47% of the PL emission couples in the HE_11_ modes, while even higher values of CD could be achieved by further enhancing the coupling efficiency or by increasing the symmetry breaking. From the application point of view, a major benefit of using III-V semiconductor NWs as the light emitting material is that they can incorporate radial PN-junctions and thus provide means for the fabrication of chiral LEDs integrated on Si platform. We believe that these results pave way for compact and integrated chiral light sources, which have a broad range of applications quantum technology, biology, and chemistry.

## Supplementary information

Supplementary Information document

## Data Availability

The datasets generated during and/or analysed during the current study are available from the corresponding author on reasonable request.
