# Publisher Correction: Demonstration of extrinsic chirality of photoluminescence with semiconductor-metal hybrid nanowires

**DOI:** 10.1038/s41598-021-86885-w

**Published:** 2021-04-01

**Authors:** Teemu Hakkarainen, Emilija Petronijevic, Marcelo Rizzo Piton, Concita Sibilia

**Affiliations:** 1 Optoelecronics Research Centre, Physics Unit, Tampere University, Korkeakoulunkatu 3, FI-33720 Tampere, Finland; 2 Department S.B.A.I., Sapienza Università di Roma, Via A. Scarpa 14, I-00161 Rome, Italy; 3 Departamento de Física, Universidade Federal de São Carlos, CP 676 São Carlos, São Paulo, Brazil

Correction to: *Scientific Reports* 10.1038/s41598-019-41615-1, published online 25 March 2019

The original version of this Article contained an error in Figure 1 where the cross-sectional transmission electron microscope micrograph was omitted in panel (d). The original Figure 1 and accompanying legend appear below.

**Figure 1:**
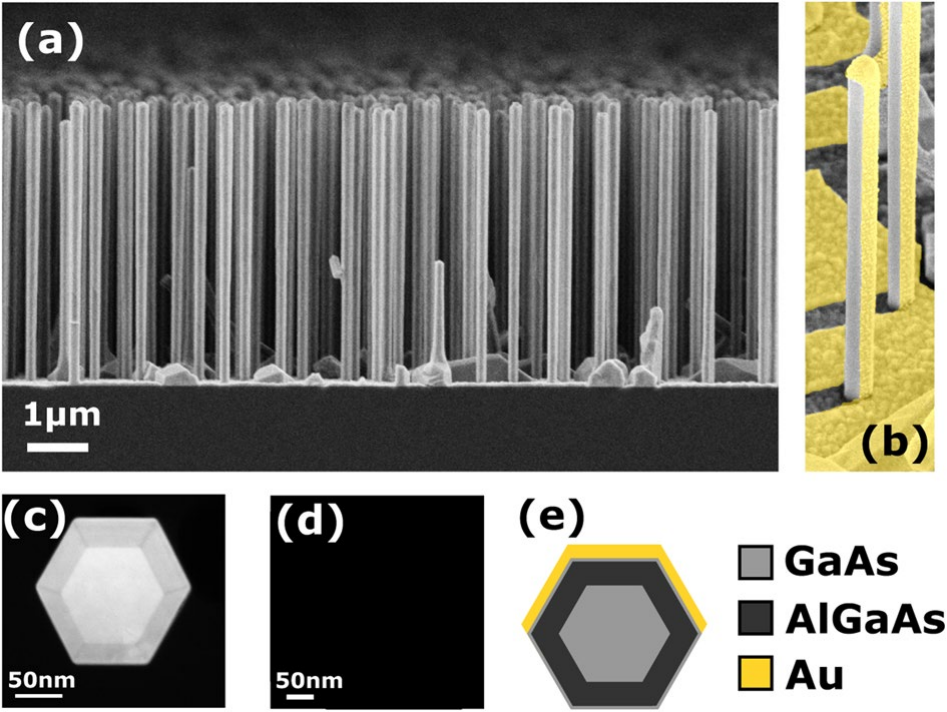
Structural details of the investigated NWs. (**a**) SEM edge view the sample with NWs 4690 ± 80 nm long, of the overall diameter 197 ± 9 nm. (**b**) False color SEM image of the NWs after Au deposition. (**c**) Cross-sectional dark-field TEM micrograph showing GaAs core, AlGaAs shell and GaAs supershell. (**d**) Cross-sectional TEM image of the NW asymmetrically covered by Au. (**e**) Cross-sectional sketch of the NW materials.

The original Article has been corrected.

