# Supplementary material for: Demonstration of extrinsic chirality of photoluminescence with semiconductor-metal hybrid nanowires
Source: Sci Rep. 2019 Mar 25;9:5040. doi: 10.1038/s41598-019-41615-1 (PMC6434037; doi:10.1038/s41598-019-41615-1)
Supplement: Supplementary file 1 — Supplementary Information document [file 41598_2019_41615_MOESM1_ESM.pdf]

Supplementary Information

Demonstration of extrinsic chirality of photoluminescence with semiconductor-metal hybrid nanowires

Teemu Hakkarainen[1,], Emilija Petronijevic[2], Marcelo Rizzo Piton[1,3], and Concita Sibilia[2]*

[1]Optoelecronics Research Centre, Physics Unit, Tampere University, Korkeakoulunkatu 3, FI-33720 Tampere, Finland

[2]Department S.B.A.I., Sapienza Università di Roma, Via A. Scarpa 14, I-00161 Rome, Italy

[3]Departamento de Física, Universidade Federal de São Carlos, CP 676 São Carlos, São Paulo, Brazil

*E-mail: teemu.hakkarainen@tuni.fi

1. Au-coated nanowires

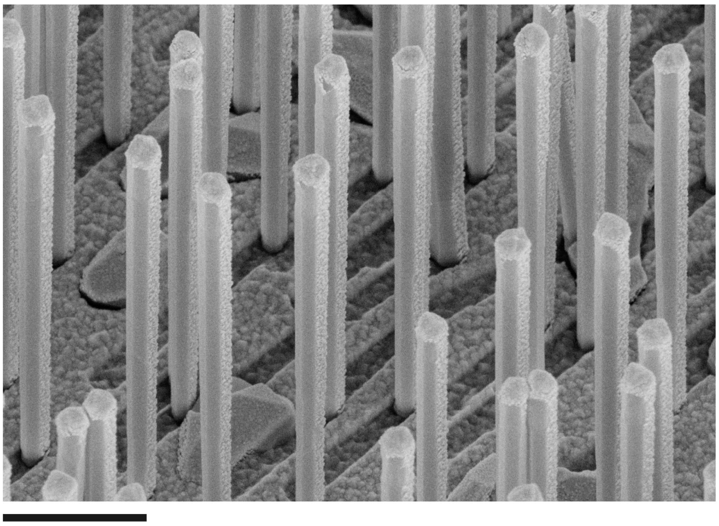

Figure S1. SEM picture showing the nanowires after the growth of the asymmetric Au-coating. The scale bar is 1 µm.

2. Experimental setup

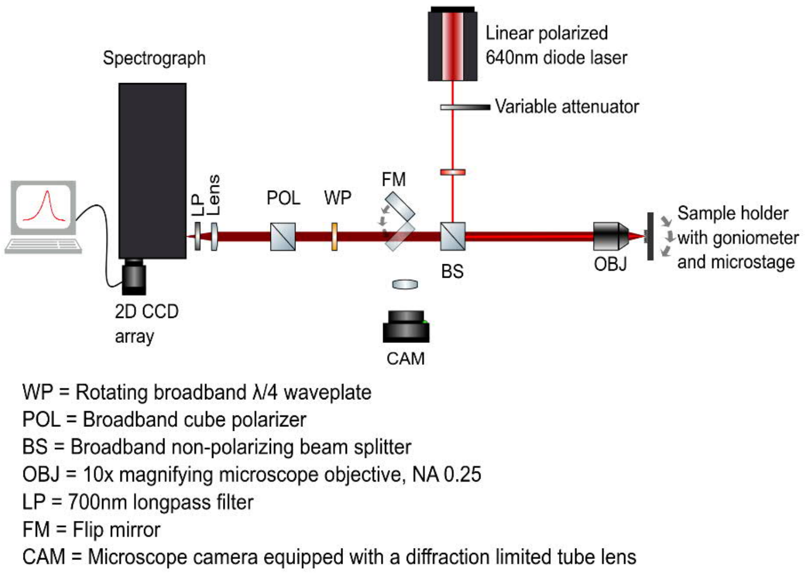

Figure S2. Illustration of the experimental setup for circular polarized photoluminescence experiments.

3. Effect of circular polarized excitation

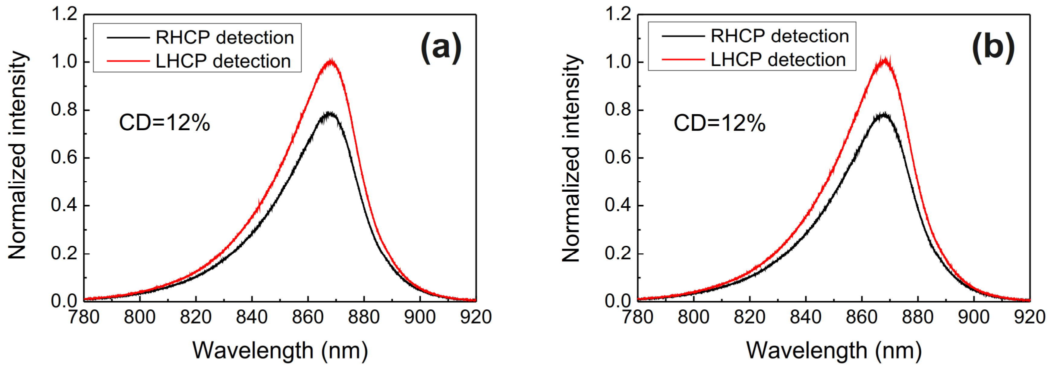

Figure S3. Effect of circular polarized excitation at 640 nm on the CD of photoluminescence emission: (a) with right-handed circular polarized (RHCP) excitation and (b) with left-handed circular polarized (LHCP) excitation, both showing CD=12% for tilt angle of 30°.

4. Linear polarization of the photoluminescence emission

A. Polarization anisotropy in the x-y plane

The linear polarization response of the photoluminescence (PL) emission for both Au-coated and Au-free NWs was measured using the experimental setup presented in Fi. S1. A broadband $\lambda/2$ waveplate was used for probing the linear polarization. The NWs were excited with a 20x microscope objective, which was used also for collecting the PL signal.

As, show in Fig. S4a, the NWs with Au show some degree of linear polarization in the direction of the Au deposition. Qualitatively, it can be expressed as the degree of linear polarization (*DOP*):

$$DOP = \frac{I_y - I_x}{I_y + I_x} \times 100\% = 5.1\%\,, \qquad \text{(S1)}$$

Where I_y and I_x are the intensities for polarizations parallel and perpendicular to the Au deposition direction, respectively. The NWs without Au do not exhibit any polarization anisotropy, as shown in Fig. S4b, as expected from the crystal symmetry of zincblende (111) direction. The slight polarization of the Au-coated NWs can be attributed to the influence of Au on the local optical density of states.

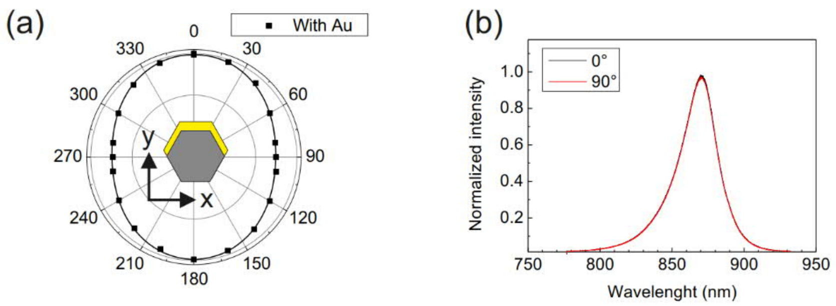

Figure S4. Linear polarization of NWs with and without Au measured in the direction parallel to the surface normal of the substrate. (a) Polar plot of normalized intensity with respect to polarization angle at GaAs peak wavelength for Au-coated NWs. The radial scale ranges from 0.5 to 1.02. The orientation of the structure is shown in the inset. (b) Linear polarized PL spectra for NWs without Au. The measurement was performed using the setup shown in Fig. S2 with a λ/2 waveplate and 10x magnifying microscope objective.

B. Polarization anisotropy: axial vs. radial

The polarization anisotropy between the axial and radial directions was measured using the experimental setup presented in Fig. S2. A broadband λ/2 waveplate was used for probing the linear polarization. A single NW transferred on a SiO_2 substrate was excited with a 40x microscope objective, which was used also for collecting the PL signal. An additional cylinder lens was place in the excitation beam path (in between the variable attenuator and beam splitter) in order to expand the excitation spot as a line which excited the whole volume of the NW. The NW was aligned in such way the emission from the whole NW was detected with the TE-cooled CCD camera.
As shown in Fig. S5, the NWs with and without Au show similar polarization along the NW axis. Qualitatively it can be expressed as the degree of linear polarization (*DOP*):

$$DOP = \frac{I_a - I_r}{I_a + I_r} \times 100\% = 23.5\%\,, \qquad \text{(S2)}$$

Where I_a and I_r are the intensities of the polarizations in the axial and radial directions, respectively. Such linear polarization is typical for (111)-oriented zincblende NWs due to the crystalline symmetry and dielectric anisotropy arising from the 1-dimensional geometry of the structure[1]. We can conclude that the Au layer does not significantly alter the emission dipole orientation in the NW.

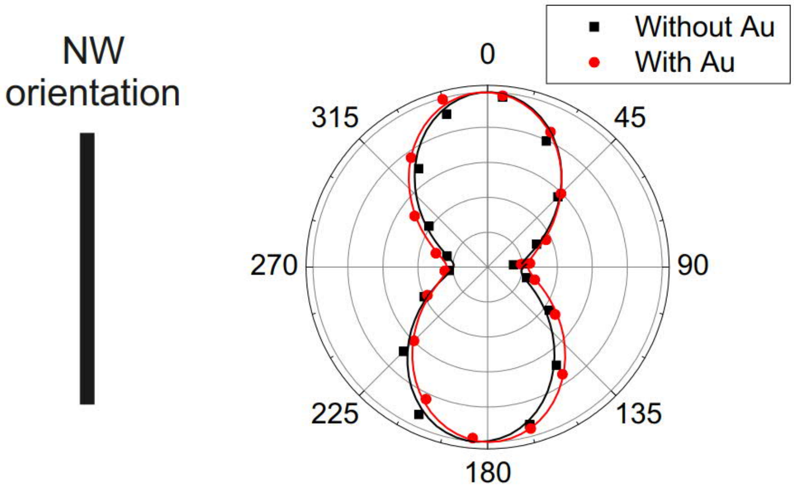

Figure S5. Linear polarization of a single NW with and without Au presented as polar plots of normalized intensity versus polarization angle at GaAs peak wavelength. The radial scale in each plot ranges from 0.5 to 1.02. The NWs were transferred on a SiO2 substrate and the measurement was performed in the direction normal to the NW sidefacet using the setup shown in Figure S1 with a λ/2 waveplate and a 40x magnifying microscope objective.

5. Nanowire density and nearest neighbor distribution

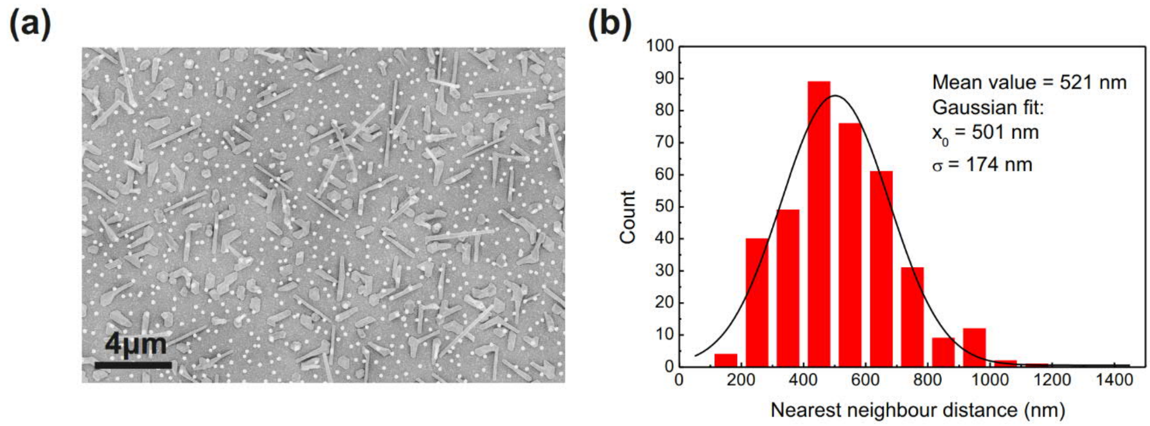

Figure S6. (a) Top-view scanning electron microscope (SEM) image of the NWs having density of $1x10^8$ cm-2. (b) Nearest neighbor distance statistics for the NWs in (a). The histogram includes the mean value of the data as well as the center (x_0) and standard deviation (σ) of the Gaussian fitting function.

6. Details on the simulation

Emission from finite NWs is governed by the coupling of the emission centers to the guided or leaky modes of the nanostructure, and it has been investigated both theoretically[2] and experimentally[3] for semiconductor NWs. We recently studied absorption properties of GaAs-based NW samples that were covered by Au by a similar process[4]: photo-acoustic spectroscopy showed that such samples still exhibit absorption resonances due to the semiconductor mode excitation, although they were much broader due to the asymmetric presence of Au. On the other hand, Au influences the confinement of the electric field, that directly governs the local density of states and hence PL. Therefore, in such structures the far field polarization and intensity of PL modelled by the dipoles is expected to highly depend on the dipole orientation and position with respect to the modal field [2].

We first use MODE solver by Lumerical to calculate the modes of infinitely long NWs, using statistically determined average values for NW dimensions, and to compare modal fields with and without the presence of Au. This tool uses the finite-difference eigenmode method to solve Maxwell's equations on a cross-sectional mesh of the waveguide, giving the effective index as an eigenvalue, and mode profile as an eigenvector. In both *x* and *y* direction the used mesh was 1 nm. Lower diameter GaAs NWs at wavelengths around 870 nm support only no-cutoff modes HE_{11x} and HE_{11y}, that are weakly guided, with the electric field confined on the borders. For the case without Au in Fig. S7a, these modes are almost degenerate with n_{HE11x}=1.557. Au layer strongly breaks the degeneracy, giving two distinct modes with n_{HE11x}=1.249+0.009i and n_{HE11y}=1.073+0.006i, with mode propagation wavelengths $\lambda_{z,HE11x}$=695nm, and $\lambda_{z,HE11y}$=809nm. Since the modes are not completely confined inside the NW (Fig. S7b), perfectly matched layers must be used for the simulation boundaries in *x* and *y* direction. Clearly, the Au layer leads to even lower confinement (the same color scale is used), and breaks the symmetry in y-direction, as can be seen especially from E_z component which is much stronger closer to Au layer. Moreover, the Au layer introduces propagation losses, as can be seen e.g. from 3D electric field intensity distribution in the NW excited with *y*-polarized field at the normal incidence, from the top. Note that for the modelling of the emission one should only consider emission from GaAs core, which effectively has a side of 65.3 nm; in Fig. S7c the electric field confinement in GaAs core follows the symmetry of the mode. Next, PL from a finite NW is modelled using Finite Difference Time Domain Solver by Lumerical. The NW and the source at 868nm are surrounded in all directions by perfect matching layers, placed at least half the wavelength from the borders of the NW to prevent from the instabilities due to the evanescent fields. A near field profile monitor placed 50nm above the NW projects the electric field components at 1 m far-field hemisphere. In order to model the experiment, these complex far fields at different angles (θ,φ) are saved as matrix, and multiplied by the Jones matrices of the lambda quarter plate and linear polarizer (Figure S7d). The matrices depend on the quarter wave plate rotation such that quarter the rotation of 45° (135°) represents right (left) handed polarization (R and L, respectively). Far-field angle θ corresponds to the tilting angle of the sample, and the azimuthal angle φ in the experiments takes values of 0 and 180° (note that φ=180° means that the sample is tilted in the negative θ direction, as in Fig. 3a in the manuscript).

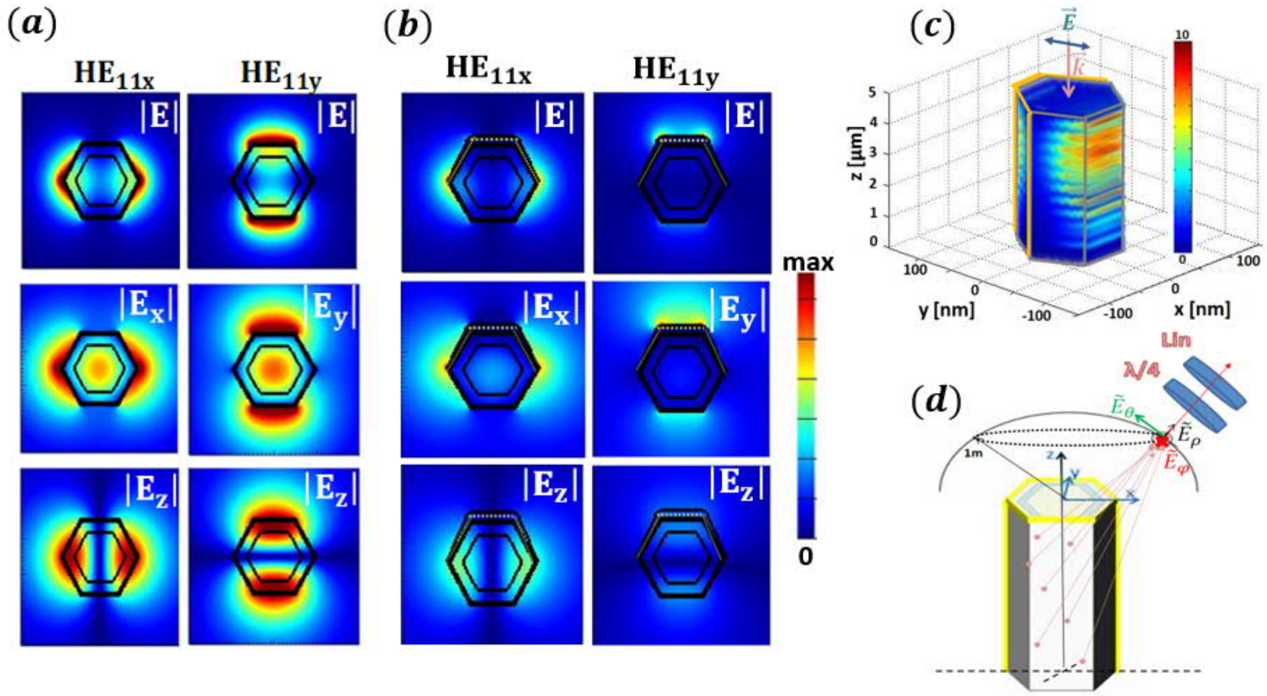

Figure S7. Electric field distribution for HE_{11x} and HE_{11y} modes for (a) the NW without Au, (b) the NW with Au. (c) 3D distribution of electric field intensity in the GaAs core. (d) Sketch of the simulation set-up with the far-field hemisphere.

Conventionally, the source of PL in the NW is modelled by placing a single point electric dipole as a radiation source at a position (*x*,*y*,*z*) inside GaAs core, and averaging the simulations over the dipole position and orientation. In Fig. S8 we show the integrated far field intensity across the whole hemisphere above the NW, as a dependence on the NW length. We see that *x*-oriented dipole has shorter oscillation wavelength than *y*-oriented dipole, in agreement with $\lambda_{HE11x} < \lambda_{HE11y}$. Further, more power give *z*-oriented, off-centered dipoles as they see stronger fields in *z*-direction; moreover, E_z is stronger closer to Au, that is why *z* dipole at (*x*=0nm, *y*=40nm) radiates more than at (*x*=0nm, *y*=-40nm). Finally, oscillations are longer for the *z*-oriented dipole when placed at (x=0nm, *y*=±40nm), than at (*x*=40nm, *y*=0nm); the first are similar to the ones of y-dipole (coupling to HE_{11y}), and the latter are similar similar to the ones of *x*-dipole (coupling to HE_{11x}). Intensity decreases due to the higher losses for longer NWs. Therefore, coupling to these different modes is highly dependent on the orientation and position of the dipoles in *xy* cross-section. Moreover, as the possible excited modes have longer propagation length than the NW length[2], one must take into account different *z* positions as they lead to the enhancement/decrease in the emission depending on the position of the antinodes/nodes of the modal field. Finally, to resolve their contribution to L or R polarization in the far field, one would need to perform high number of simulations to account for all these properties.

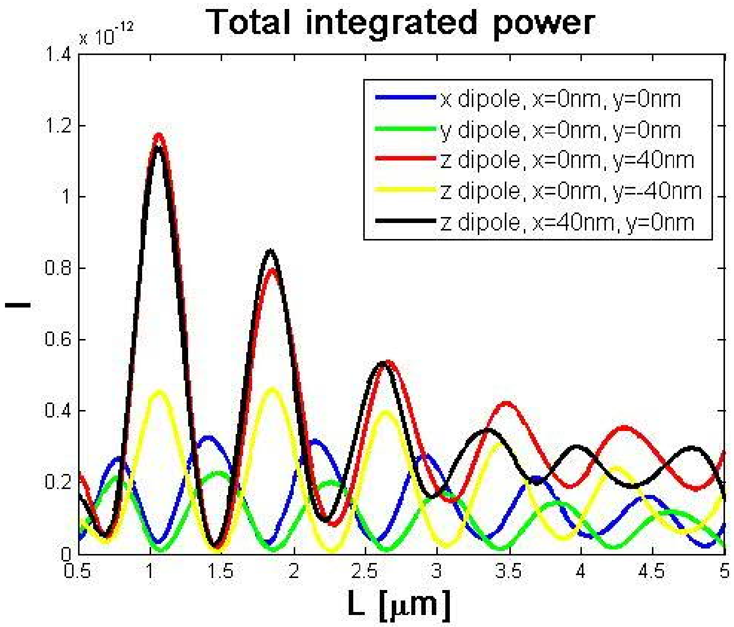

Figure S8. Total integrated far-field intensity as the function of NW length.

In the manuscript, instead, we use the complex modal fields saved from MODE simulations to import them as sources for FDTD simulations. Such fields contain field distributions of all three electric field components across the NW cross section, and propagation properties in *z*-direction. The first simplifies the issue of averaging over different dipole orientations, and the latter simplifies the averaging of the dipole positions in *z*-direction. Therefore, only two simulations are needed to account for the far-field properties when the NW is excited with HE_{11x} or HE_{11y} mode. Without Au, these modes have no losses and lead to similar emission in the far-field, as shown in Fig. S9. By applying the Jones matrices to the complex fields at each point in the far-field, we calculate the contribution to L or R polarization of the mode. As expected, for such symmetric case the difference between L and R cancels out for both modes everywhere at φ=0°, 90°, 180° and 270°, giving small differences in regions between these angles. These differences are of the opposite sign for HE_{11x} and HE_{11y}; as the emission is a result of the coupling to the both modes, we finally consider the intensity emitted to L and R from the both modes. Finally, the difference between L and R for the sum of the modes gives no chiral behavior at all far-field points.

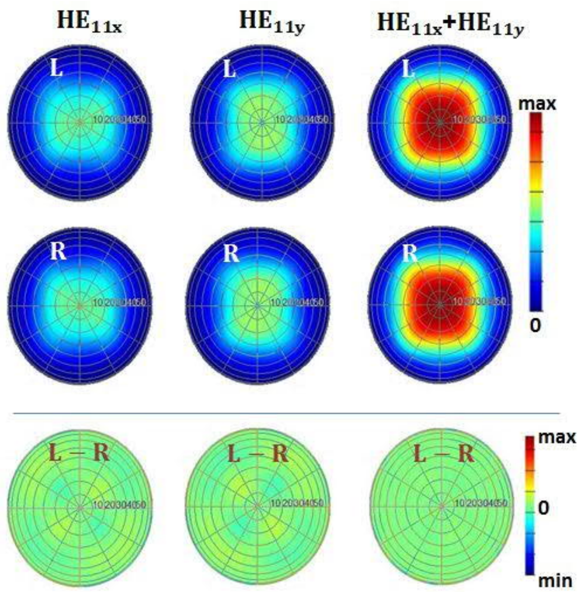

Figure S9. Simulated left-handed (L) and right-handed (R) polarizations of the far-field intensities emitted from HE_{11x} and HE_{11y} modes emitted from the NW without Au. The tilt axis θ in ranges from 0° to 90° with 10° steps while the azimuthal axis φ presents a full rotation around the NW axis.

The modes are strongly affected by the introduction of the asymmetric Au layer: HE_{11x} has higher losses and symmetric electric field intensity in the core, while for HE_{11y} the field is stronger closer to the Au layer. This directly influences the far-field polarization, as shown in Fig. S10. HE_{11x} mode gives lower overall intensity for both L and R polarizations, and their difference shows no chiral behavior for all θ at φ=0°, 90°, 180° and 270°, while there is a negligible chirality elsewhere. HE_{11y} mode has lower losses and gives higher intensity in both polarizations; the difference between these components that leads to CD is anti-symmetric for φ=0° and φ=180°, and 0 for φ=90° and φ=270°, which is characteristic for extrinsic chirality. Finally, the overall intensity of two modes is governed by the asymmetry of HE_{11y} mode, and the difference maximum exactly matches the experimentally observed angle of maximum CD, i.e. θ=20° (φ=0°).

In order to fit experimental CD, we consider contributions from both modes as well as the uncoupled part of the emission that behaves as a Lambertian source:

$$CD = 100 \cdot \frac{(I_{HE_{11x},L} + I_{HE_{11y},L} + I_{Lam} cos\theta) - (I_{HE_{11x},R} + I_{HE_{11y},R} + I_{Lam} cos\theta)}{I_{HE_{11x},L} + I_{HE_{11y},L} + I_{HE_{11x},R} + I_{HE_{11y},R} + 2 I_{Lam} cos\theta}, \qquad (S3)$$

where $I_{HE11i,j}$ stands for the intensity emitted from HE_{11i} mode to j polarization, i=(x,y), j=(L,R), and I_{Lam} is the maximum intensity of the Lambertian source. I_{Lam} is used as the only fitting parameter to obtain CD value from the experiment, and calculate the coupling efficiency as explained in the main manuscript.

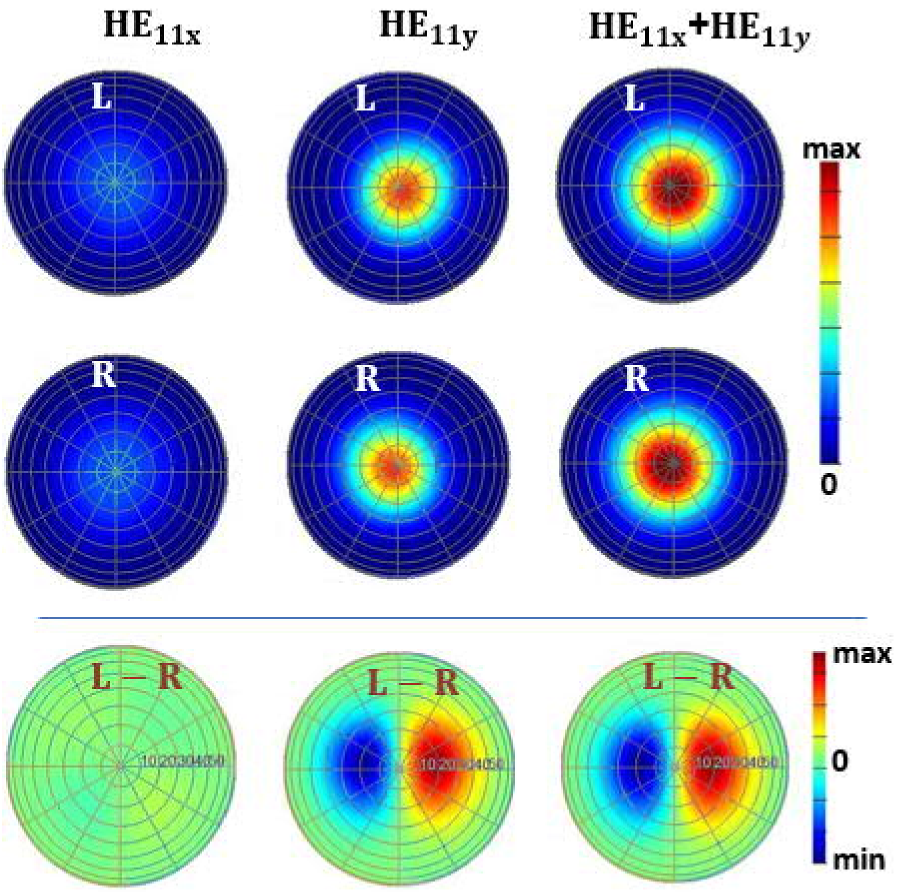

Figure S10. Simulated left-handed (L) and right-handed (R) polarizations of the far-field intensities emitted from HE_{11x} and HE_{11y} modes emitted from the NW with Au. The tilt axis θ ranges from 0° to 90° with 10° steps while the azimuthal axis φ presents a full rotation around the NW axis.

From previous dipole source simulations we noted that single point dipoles can provide higher CD, that is lowered by averaging the contributions from the various dipoles across the volume. In Fig. S11 we show the results averaged over three dipole orientations for dipoles placed in the NW center, that gives three times higher CD at the angle θ=12° (note that high CD on the borders of the graph is due to numerical errors, and should not be considered as there is no intensity emitted at these angles). Therefore, confining the emitters to a small volume can enhance CD and change its angular behavior.

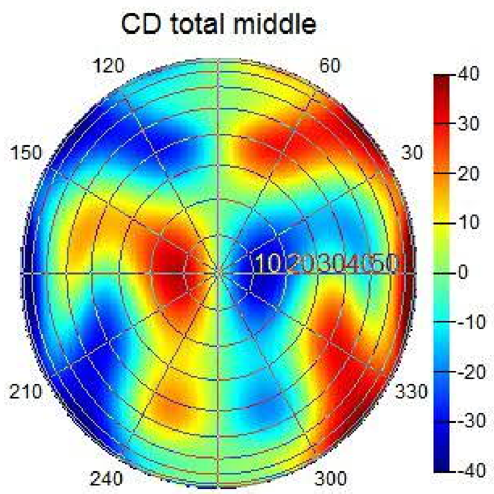

Figure S11. CD of the dipole placed at the NW center and averaged over three directions.

References

1. Wilhelm, C., Larrue, A., Dai, X., Migas, D. & Soci, C. Anisotropic photonic properties of III–V nanowires in the zinc-blende and wurtzite phase. *Nanoscale* **4,** 1446 (2012).

2. Paniagua-Domínguez, R., Grzela, G., Rivas, J. G., & Sánchez-Gil, J. A. Enhanced and directional emission of semiconductor nanowires tailored through leaky/guided modes. *Nanoscale* **5**, 10582 (2013).

3. Abujetas, D. R., Paniagua-Domíguez, R. & Sanchez-Gil, J. A. Unraveling the janus role of mie resonances and leaky/guided modes in semiconductor nanowire absorption for enhanced light harvesting. *ACS Photonics* **2**, 921 (2015).

4. Leahu *et al.* Evidence of optical circular dichroism in GaAs-based nanowires partially covered with gold. *Adv. Opt. Mater.* **5**, 1601063 (2017).
